# Differential impact of overt and subclinical hypothyroidism on severe postpartum hemorrhage: a retrospective cohort study

**DOI:** 10.3389/fendo.2025.1654856

**Published:** 2025-11-05

**Authors:** Jungou Zhuang, Qiaoli Guo, Chenning Liu, Yujiao Fu, Yuhui Liu, Li Tang, Fang He

**Affiliations:** ^1^ Department of Obstetrics and Gynecology, Dongguan Maternal and Child Health Care Hospital, Dongguan, Guangdong, China; ^2^ The First School of Clinical Medicine, Southern Medical University, Guangzhou, Guangdong, China; ^3^ State Key Laboratory of Quality Research in Chinese Medicine, Institute of Chinese Medical Sciences, University of Macau, Macao, Macao SAR, China; ^4^ Department of Obstetrics and Gynecology, Guangdong Provincial Key Laboratory of Major Obstetric Diseases, Guangdong Provincial Clinical Research Center for Obstetrics and Gynecology, Guangzhou Medical University, Guangzhou, China; ^5^ Guangdong-Hong Kong-Macao Greater Bay Area Higher Education Joint Laboratory of Maternal-Fetal Medicine, The Third Affiliated Hospital, Guangzhou Medical University, Guangzhou, China

**Keywords:** hypothyroidism, severe postpartum hemorrhage, subclinical hypothyroidism, overt hypothyroidism, risk factors

## Abstract

**Background:**

The impact of hypothyroidism, which can be divided into overt hypothyroidism and subclinical hypothyroidism, on severe postpartum hemorrhage (SPPH) remains unknown.

**Methods:**

A total of 34,303 pregnant women from the Third Affiliated Hospital of Guangzhou Medical University between 2016 and 2020 were included in this retrospective cohort study. We employed three logistic regression models incorporating different covariates to explore the relationship between hypothyroidism and SPPH, followed by an interaction analysis to identify potential modifiers. Then, we performed a stratified analysis to examine the influence of potential modifiers on the association between hypothyroidism and SPPH. Finally, sensitivity analyses were conducted to evaluate the robustness of our findings.

**Results:**

In the logistic regression analysis, we found that hypothyroidism was correlated with an elevated risk of SPPH (OR = 1.609, 95% CI: 1.111–2.329, *p* = 0.012). In addition, overt hypothyroidism had a significant impact on the increased risk of SPPH (OR = 1.688, 95% CI: 1.137–2.507, *p* = 0.009), whereas no significant association with SPPH was observed for subclinical hypothyroidism (OR = 1.208, 95% CI: 0.443–3.291, *p* = 0.712). In the relationship between hypothyroidism and SPPH, we observed that age and history of radiation exposure acted as potential modifiers (*p* for interaction < 0.05). Additionally, the correlation between hypothyroidism and SPPH was stronger in pregnant women aged < 35 years (OR = 2.412, 95% CI: 1.583–3.673, *p* < 0.001) than in those aged ≥ 35 years (OR = 0.755, 95% CI: 0.364–1.567, *p* = 0.450).

**Conclusions:**

Overt hypothyroidism had a significant impact on the elevated risk of SPPH, especially in pregnant women aged < 35 years. In contrast, subclinical hypothyroidism showed no significant association with SPPH, which may reflect limited statistical power rather than the absence of risk. These findings offer valuable insights into the relationship between hypothyroidism and SPPH, potentially optimizing maternal outcomes by preventing and intervening in the occurrence of SPPH.

## 1 Introduction

Postpartum hemorrhage (PPH), a major contributor to maternal mortality and severe morbidity, accounts for nearly a third of the deaths among pregnant and postpartum women (1, 2). Severe postpartum hemorrhage (SPPH) can lead to complications such as hemorrhagic shock, disseminated intravascular coagulation, acute renal failure, loss of fertility, pituitary necrosis (Sheehan syndrome), and even maternal or neonatal death (3). In recent years, the incidence of PPH incidence has been on the rise, with an increase of at least 26% over the past decade in the United States (4).

Maternal hypothyroidism is a common endocrine disorder of pregnancy, with a steadily increasing incidence (5). Overt hypothyroidism is estimated to affect 0.3–1.0% of pregnancies, while subclinical hypothyroidism ranges from 4.0% to 17.8% (6). Hypothyroidism, both overt and subclinical, have been associated with adverse maternal outcomes, including placental abruption (7, 8), polyhydramnios (8, 9), gestational diabetes mellitus (5, 7), and premature rupture of membranes (5, 10).

However, the relationship between hypothyroidism and PPH, particularly SPPH, is less frequently discussed. Some studies suggested that pregnant women with hypothyroidism had a higher risk of PPH. A Canadian study (5), involving more than 18,400 participants, reported that women with hypothyroidism were more likely to experience PPH during labor. Similarly, Kiran et al. (11) showed a significant association between PPH and hypothyroidism. On the contrary, Gur et al. (12) and Wang et al. (13) indicated that no statistical difference was observed in the incidence of PPH between hypothyroidism and euthyroid pregnant women. In these studies, PPH was defined as a blood loss of 500 mL or more within 24 hours after birth. The research data on hypothyroidism and SPPH remain very limited.

Thus, we aimed to investigate the relationship between hypothyroidism (including overt and subclinical hypothyroidism) and SPPH through a retrospective cohort study, utilizing data from the institutional medical record database of all women delivering at the Third Affiliated Hospital of Guangzhou Medical University from 2016 to 2020. This work could offer valuable insights into the influence of hypothyroidism on SPPH, potentially optimizing maternal outcomes by preventing and intervening in its occurrence.

## 2 Materials and methods

### 2.1 Study participants

**Figure 1:**
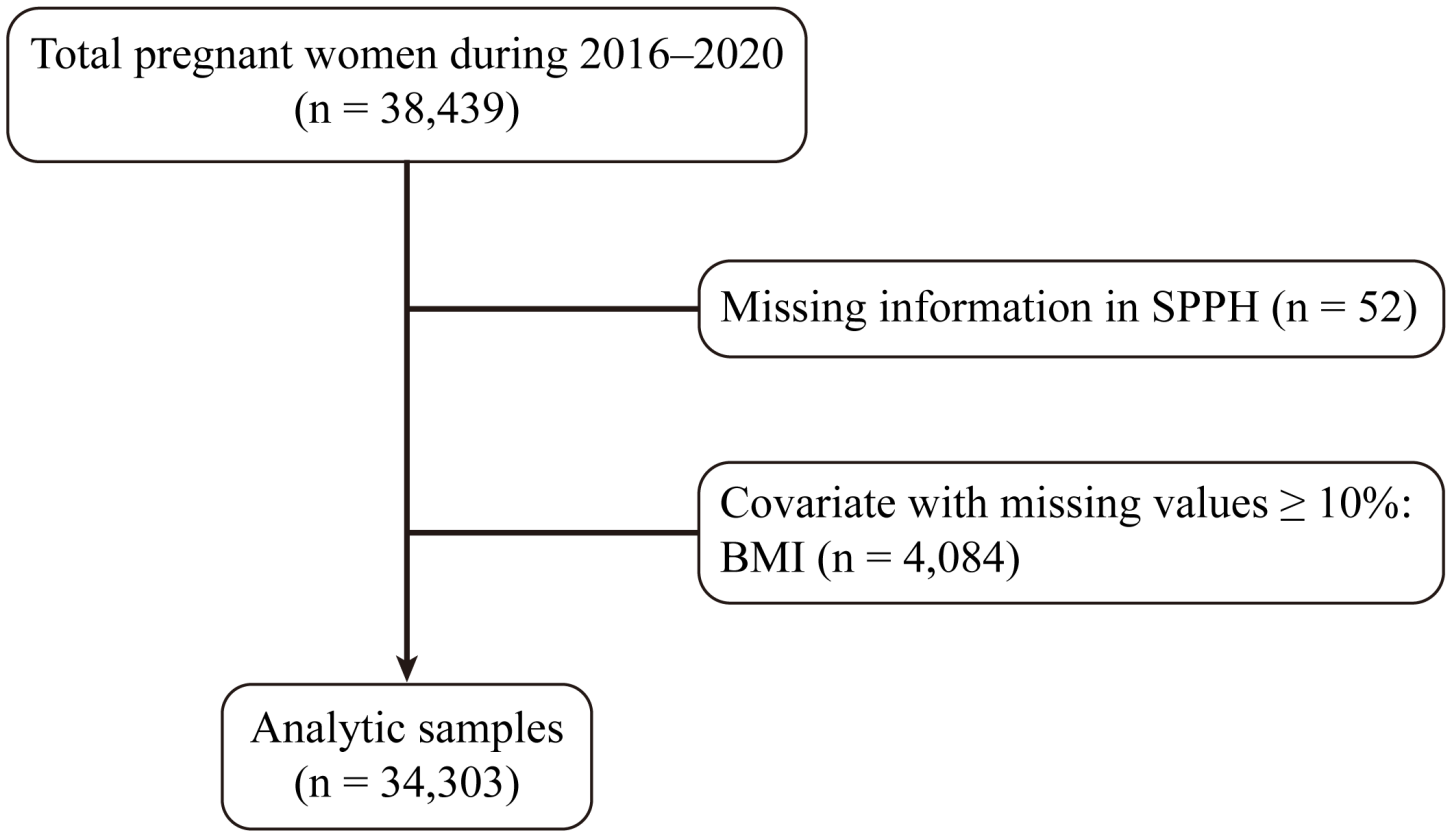
Flowchart of the study design. SPPH, severe postpartum hemorrhage; BMI, body mass index.

Ethical approval (approval number: [2024] 142) was obtained for this analysis and each participant signed informed consent. This study examined the institutional database encompassing all women who gave birth at the Third Affiliated Hospital of Guangzhou Medical University (Guangzhou Medical Centre for Critical Pregnant Women) from January 2016 to December 2020. In total, 38,439 women were included in the analysis. Women lacking SPPH information (n = 52) were excluded from the analysis. Additionally, pregnancies with missing values comprising ≥ 10% of covariates, such as pregestational body mass index (BMI) (n = 4,084), were removed. Subsequently, missing values for covariates comprising < 10% were imputed using the random forest method. Thus, our final analysis included a total of 34,303 pregnant women (**Figure 1**).

### 2.2 Definitions

The outcome of the research focused on SPPH, defined as an estimated hemorrhage of ≥ 1,000 mL within 24 hours after childbirth. Blood loss assessment was based on a combination of visual estimation, gravimetric method, estimated blood loss volume, shock index, and hemoglobin levels, as documented by the attending healthcare providers, including physicians, midwives, and nurses. The final estimated value was recorded by the resident physician who attended the delivery.

The diagnosis of hypothyroidism during pregnancy was based on the Guidelines on Diagnosis and Management of Thyroid Diseases during Pregnancy and Postpartum (14, 15). Overt hypothyroidism was defined as serum thyroid-stimulating hormone (TSH) levels above the trimester-specific reference ranges with free thyroxine (FT4) levels below the corresponding lower limit, while subclinical hypothyroidism was defined as elevated TSH levels with normal FT4. Measurements of TSH and FT4 were performed employing a chemiluminescent immunoassay with reagents from Abbott Laboratories. Trimester-specific reference ranges are shown in **Supplementary Tables 1**, **2**. Laboratory test reports from hospitals of equivalent level were also acceptable. Cases of hypothyroidism were ascertained using the corresponding International Classification of Diseases (ICD) codes from the electronic health records. Regardless of thyroid peroxidase antibody (TPOAb) status (positive or negative), all pregnant women diagnosed with hypothyroidism routinely received levothyroxine (LT4) therapy, with dose adjustment to maintain TSH within the guideline-recommended levels.

### 2.3 Covariates

To reduce potential biases in our analysis, we chose covariates based on previous research findings and clinical plausibility. The analysis incorporated the following covariates: age, pregestational body mass index (BMI), education level, history of radiation exposure, previous cesarean delivery, number of fetuses, gestational age, birth weight, head circumference of newborns, stillbirth, hypertensive disorders of pregnancy, HELLP syndrome, diabetes, intrahepatic cholestasis of pregnancy (ICP), anemia, thrombocytopenia, placenta previa, placental abruption, placenta accreta spectrum (PAS), polyhydramnios, fetal anomalies, malposition, cesarean delivery, prolonged second stage of labor, and fetal distress. Full list of covariates investigated in analysis are provided in **Supplementary Table 3**.

### 2.4 Statistical analysis

Continuous data with a normal distribution were presented as mean (± standard deviation [SD]) and were compared using the independent samples t-test. Variables with skewed distributions were expressed as medians [first quartile (P25) and third quartile (P75)] and were compared using the nonparametric Wilcoxon rank-sum test. Categorical variables were expressed as percentages (%) and were compared using either the chi-square test or Fisher’s exact test, as appropriate. We utilized three logistic regression models to explore the impact of overt or subclinical hypothyroidism on SPPH. Model 1 did not adjust for any covariates. Model 2 made adjustments for age, pregestational BMI, education level, history of radiation exposure, previous cesarean delivery, number of fetuses, gestational age, and birth weight. Model 3 further adjusted for head circumference of newborns, stillbirth, hypertensive disorders of pregnancy, HELLP syndrome, diabetes, ICP, anemia, thrombocytopenia, placenta previa, placental abruption, PAS, polyhydramnios, fetal anomalies, malposition, cesarean delivery, prolonged second stage of labor, and fetal distress based on Model 2. Subsequently, we conducted an interaction analysis, finding that age and history of radiation exposure acted as potential modifiers in the relationship between hypothyroidism and SPPH. Considering the relatively small number of patients with SPPH in the history of radiation exposure, stratified analysis was done only for age. To examine the influence of age on the association between hypothyroidism and SPPH, we stratified the study cohort based on age, employing a cut-off threshold of 35 years. Furthermore, sensitivity analyses were conducted to evaluate the robustness of our findings; In one analysis, participants who delivered twins or triplets were excluded, while in the other sensitivity analysis, participants with a history of radiation exposure and an unknown radiation exposure history were removed. All *p* values reported were two-sided, with a significance level established at 0.05. Statistical analyses were performed using IBM SPSS Statistics 26.

## 3 Results

### 3.1 Population characteristics

The baseline characteristics of the study population are shown in **Table 1**. In our analysis, we included 34,303 pregnant women, among whom 913 were diagnosed with SPPH, and the average age of the participants with SPPH was 32.77 ± 4.84 years. Overweight women (pregestational BMI exceeding 23.9) were more frequently observed in the SPPH group. Low educational level, history of radiation exposure, and previous cesarean delivery were more common among pregnant women with SPPH. Furthermore, the SPPH group exhibited higher rates of hypertensive disorders of pregnancy, diabetes, anemia, thrombocytopenia, placenta previa, placental abruption, PAS, cesarean delivery, prolonged second stage of labor, and hypothyroidism. Baseline characteristics stratified by thyroid status are provided in **Supplementary Table 4**.

**Table 1:** Baseline characteristics of study participants.

Variables	Overall (n = 34,303)	SPPH (n = 913)	Non-SPPH (n = 33,390)	*P*-value
Age (years)	31.24 ± 4.78	32.77 ± 4.84	31.20 ± 4.77	< 0.001
Pregestational BMI (kg/m^2^), n (%)				0.036
< 18.5	2,164 (6.31%)	51 (5.58%)	2,113 (6.33%)	
18.5~23.9	17,173 (50.06%)	428 (46.88%)	16,745 (50.15%)	
24.0~27.9	10,166 (29.64%)	280 (30.67%)	9,886 (29.61%)	
≥ 28	4,800 (13.99%)	154 (16.87%)	4,646 (13.91%)	
Education level, n (%)				< 0.001
College or above	23,484 (68.46%)	501 (54.87%)	22,983 (68.83%)	
High school or equivalent	4,063 (11.85%)	126 (13.80%)	3,937 (11.79%)	
Middle school	6,226 (18.15%)	244 (26.73%)	5,982 (17.92%)	
Less than middle school	530 (1.54%)	42 (4.60%)	488 (1.46%)	
History of radiation exposure, n (%)	129 (0.38%)	3 (0.33%)	126 (0.38%)	1.000
Previous cesarean delivery, n (%)				< 0.001
0	27,561 (80.35%)	497 (54.44%)	27,064 (81.05%)	
1	6,023 (17.56%)	295 (32.31%)	5,728 (17.16%)	
≥ 2	719 (2.09%)	121 (13.25%)	598 (1.79%)	
Number of fetuses, n (%)				< 0.001
Singleton	31,904 (93.01%)	816 (89.37%)	31,088 (93.11%)	
Twin	2,360 (6.88%)	91 (9.97%)	2,269 (6.79%)	
Triplet	39 (0.11%)	6 (0.66%)	33 (0.10%)	
Gestational age (weeks)	37.78 ± 3.30	36.31 ± 3.57	37.82 ± 3.28	< 0.001
Birth weight^a^ (g)	2,991.74 ± 697.72	2,797.51 ± 776.50	2,997.05 ± 694.69	< 0.001
Head circumference of newborns (cm)	32.53 ± 2.79	32.31 ± 3.41	32.53 ± 2.77	0.015
Stillbirth^b^, n (%)	1,096 (3.20%)	37 (4.05%)	1,059 (3.17%)	0.135
Hypertensive disorders of pregnancy, n (%)	2,474 (7.21%)	90 (9.86%)	2,384 (7.14%)	0.002
HELLP syndrome, n (%)	54 (0.16%)	3 (0.33%)	51 (0.15%)	0.369
Diabetes, n (%)	6,364 (18.55%)	221 (24.21%)	6,143 (18.40%)	< 0.001
ICP, n (%)	337 (0.98%)	5 (0.55%)	332 (0.99%)	0.177
Anemia, n (%)	7,439 (21.69%)	447 (48.96%)	6,992 (20.94%)	< 0.001
Thrombocytopenia, n (%)	213 (0.62%)	11 (1.21%)	202 (0.61%)	0.023
Placenta previa, n (%)	1,306 (3.81%)	382 (41.84%)	924 (2.77%)	< 0.001
Placental abruption, n (%)	423 (1.23%)	21 (2.30%)	402 (1.20%)	0.003
PAS, n (%)	1,078 (3.14%)	347 (38.01%)	731 (2.19%)	< 0.001
Polyhydramnios, n (%)	220 (0.64%)	9 (0.99%)	211 (0.63%)	0.186
Fetal anomalies, n (%)	502 (1.46%)	12 (1.31%)	490 (1.47%)	0.704
Malposition, n (%)	1,793 (5.23%)	60 (6.57%)	1,733 (5.19%)	0.064
Cesarean delivery, n (%)	13,682 (39.89%)	564 (61.77%)	13,118 (39.29%)	< 0.001
Prolonged second stage of labor, n (%)	254 (0.74%)	19 (2.08%)	235 (0.70%)	< 0.001
Fetal distress, n (%)	1,961 (5.72%)	55 (6.02%)	1,906 (5.71%)	0.685
Hypothyroidism, n (%)	980 (2.86%)	39 (4.27%)	941 (2.82%)	0.009
Subclinical hypothyroidism	154 (0.45%)	5 (0.55%)	149 (0.45%)	
Overt hypothyroidism	826 (2.41%)	34 (3.72%)	792 (2.37%)	

a The weights of twins and triplets were averaged over all fetuses.

b Stillbirth is defined as the death of at least one fetus.

SPPH, severe postpartum hemorrhage; BMI, body mass index; ICP, intrahepatic cholestasis of pregnancy; PAS, placenta accreta spectrum.

### 3.2 Impact of overt and subclinical hypothyroidism on SPPH

**Table 2** illustrates the association between hypothyroidism and SPPH using three logistic regression models. In model 1, a positive association between hypothyroidism and SPPH was observed (odds ratio [OR] = 1.539, 95% confidence interval [CI]: 1.109–2.134, *p* = 0.009). Upon dividing hypothyroidism into overt hypothyroidism and subclinical hypothyroidism, it was found that compared to controls, the OR for SPPH with overt hypothyroidism and subclinical hypothyroidism were 1.594 (95% CI: 1.123–2.261, *p* = 0.009) and 1.246 (95% CI: 0.510–3.045, *p* = 0.630), respectively. After adjusting for age, pregestational BMI, education level, history of radiation exposure, previous cesarean delivery, number of fetuses, gestational age and birth weight in model 2, it was found that the association between hypothyroidism and increased SPPH risk persisted (OR = 1.477, 95% CI: 1.056–2.066, *p* = 0.023). Consistent with the findings of model 1, overt hypothyroidism was associated with an increased risk of SPPH (OR = 1.587, 95% CI: 1.109–2.271, *p* = 0.012) while subclinical hypothyroidism didn’t have a prominent impact on SPPH (OR = 0.993, 95% CI: 0.397–2.483, *p* = 0.988), compared to controls. Moreover, model 3 further adjusted for head circumference of newborns, stillbirth, hypertensive disorders of pregnancy, HELLP syndrome, diabetes, ICP, anemia, thrombocytopenia, placenta previa, placental abruption, PAS, polyhydramnios, fetal anomalies, malposition, cesarean delivery, prolonged second stage of labor, and fetal distress based on model 2. Subsequently, we observed a consistent finding that hypothyroidism (OR = 1.609, 95% CI: 1.111–2.329, *p* = 0.012), particularly overt hypothyroidism (OR = 1.688, 95% CI: 1.137–2.507, *p* = 0.009), was correlated with an elevated risk of SPPH. Nevertheless, subclinical hypothyroidism showed no significant association with SPPH.

**Table 2:** Association between overt/subclinical hypothyroidism and SPPH by logistic models^a^.

Study Groups	Model 1^b^	Model 2^b^	Model 3^b^
Hypothyroidism (n = 980)	1.539 (1.109, 2.134)^**^	1.477 (1.056, 2.066)^*^	1.609 (1.111, 2.329)^*^
Controls (n = 33,323)	Ref.	Ref.	Ref.
Subclinical hypothyroidism (n = 154)	1.246 (0.510, 3.045)	0.993 (0.397, 2.483)	1.208 (0.443, 3.291)
Overt hypothyroidism (n = 826)	1.594 (1.123, 2.261)^**^	1.587 (1.109, 2.271)^*^	1.688 (1.137, 2.507)^**^

a The associations between Overt/subclinical hypothyroidism and SPPH are presented as ORs and 95% CIs. ^*^ *p* < 0.05; ^**^ *p* < 0.01.

b Model 1 did not adjust for any covariates. In model 2, adjustments were made for age, pregestational BMI, education level, history of radiation exposure, previous cesarean delivery, number of fetuses, gestational age, and birth weight. Model 3 further adjusted for head circumference of newborns, stillbirth, hypertensive disorders of pregnancy, HELLP syndrome, diabetes, ICP, anemia, thrombocytopenia, placenta previa, placental abruption, PAS, polyhydramnios, fetal anomalies, malposition, cesarean delivery, prolonged second stage of labor, and fetal distress based on Model 2.

SPPH, severe postpartum hemorrhage; ORs, odds ratios; CIs, confidence intervals; BMI, body mass index; ICP, Intrahepatic cholestasis of pregnancy; PAS, placenta accreta spectrum.

### 3.3 Stratified analysis

We conducted an interaction analysis and found that age and history of radiation exposure acted as potential modifiers in the relationship between hypothyroidism and SPPH (*p* for interaction < 0.05). Due to the relatively small number of patients with SPPH in the history of radiation exposure, stratified analysis was performed only for age. **Table 3** indicates the correlation between hypothyroidism and SPPH stratified by age, using 35 years as the cut-off (*p* for interaction < 0.05). The ORs were stronger in women aged < 35 years (OR = 2.412, 95% CI: 1.583–3.673, *p* < 0.001) compared to those aged ≥ 35 years (OR = 0.755, 95% CI: 0.364–1.567, *p* = 0.450). For participants aged < 35 years, the ORs for SPPH with overt hypothyroidism and subclinical hypothyroidism were 2.492 (95% CI: 1.585–3.916, *p* < 0.001) and 2.006 (95% CI: 0.671–5.996, *p* = 0.213), respectively. For women aged ≥ 35 years, the ORs for SPPH with overt and subclinical hypothyroidism were 0.807 (95% CI: 0.372–1.749, *p* = 0.586) and 0.487 (95% CI: 0.058–4.119, *p* = 0.509), respectively.

**Table 3:** Stratified analysis in the relationship between hypothyroidism and SPPH by logistic models^a^.

Study Groups	Age < 35 (n = 25,937)	Age ≥ 35 (n = 8,366)
Hypothyroidism	2.412 (1.583, 3.673)^***^	0.755 (0.364, 1.567)
Controls	Ref.	Ref.
Subclinical hypothyroidism	2.006 (0.671, 5.996)	0.487 (0.058, 4.119)
Overt hypothyroidism	2.492 (1.585, 3.916)^***^	0.807 (0.372, 1.749)

a The associations between Overt/subclinical hypothyroidism and SPPH stratified by age are presented as ORs and 95% CIs. ^***^ *p* < 0.001. To investigate the effect of age in the relationship between hypothyroidism and SPPH, the study population were stratified by age. Model adjusted for pregestational BMI, education level, history of radiation exposure, previous cesarean delivery, number of fetuses, gestational age, birth weight, head circumference of newborns, stillbirth, hypertensive disorders of pregnancy, HELLP syndrome, diabetes, ICP, anemia, thrombocytopenia, placenta previa, placental abruption, PAS, polyhydramnios, fetal anomalies, malposition, cesarean delivery, prolonged second stage of labor, and fetal distress.

SPPH, severe postpartum hemorrhage; ORs, odds ratios; CIs, confidence intervals; BMI, body mass index; ICP, Intrahepatic cholestasis of pregnancy; PAS, placenta accreta spectrum.

### 3.4 Sensitivity analyses

We conducted several sensitivity analyses to verify the stability of our results (**Table 4**). In one sensitivity analysis, we excluded participants who delivered twins or triplets. Additionally, another sensitivity analysis eliminated participants with a history of radiation exposure and those with an unknown radiation exposure history. The sensitivity analyses indicated that overt hypothyroidism remained a substantial influence on elevating the risk of SPPH, whereas subclinical hypothyroidism was not found to exert a notable impact on SPPH.

**Table 4:** Sensitivity analyses in the relationship between hypothyroidism and SPPH by logistic models^a^.

Study Groups	Singleton (n = 35,667)	Non-History of Radiation Exposure (n = 20,866)
Hypothyroidism	1.429 (0.938, 2.176)	2.564 (1.592, 4.130)^***^
Controls	Ref.	Ref.
Subclinical hypothyroidism	0.772 (0.207, 2.873)	2.692 (0.793, 9.134)
Overt hypothyroidism	1.557 (1.001, 2.421)^*^	2.543 (1.523, 4.248)^***^

a The associations between Overt/subclinical hypothyroidism and SPPH in singleton and non-history of radiation exposure are presented as ORs and 95% CIs. ^*^ *p* < 0.05; ^***^ *p* < 0.001. Model adjusted for age, pregestational BMI, education level, history of radiation exposure, previous cesarean delivery, number of fetuses, gestational age, birth weight, head circumference of newborns, stillbirth, hypertensive disorders of pregnancy, HELLP syndrome, diabetes, ICP, anemia, thrombocytopenia, placenta previa, placental abruption, PAS, polyhydramnios, fetal anomalies, malposition, cesarean delivery, prolonged second stage of labor, and fetal distress.

SPPH, severe postpartum hemorrhage; ORs, odds ratios; CIs, confidence intervals; BMI, body mass index; ICP, Intrahepatic cholestasis of pregnancy; PAS, placenta accreta spectrum.

## 4 Discussion

In this retrospective cohort study, we had several important findings. First, we observed an elevated risk of SPPH in pregnant women with hypothyroidism. Second, we found that overt hypothyroidism was significantly associated with an elevated risk of SPPH, whereas no significant association with SPPH was detected for subclinical hypothyroidism. Finally, we observed that age and history of radiation exposure acted as potential modifiers in the relationship between hypothyroidism and SPPH. Moreover, the correlation between hypothyroidism and SPPH was stronger in pregnant women aged < 35 years than in those aged ≥ 35 years.

Multiple studies demonstrated that hypothyroidism was associated with adverse pregnancy outcomes (16–19), including preterm delivery, stillbirth, cesarean delivery, and PPH (5, 17), and perinatal complications such as abortion, gestational diabetes mellitus, and gestational hypertension (20–22). Moreover, overt hypothyroidism is associated with an increased risk of preterm complications and adverse pregnancy outcomes such as abortion, gestational diabetes mellitus, gestational hypertension, preeclampsia, preterm delivery, and cesarean delivery (9, 23–29). However, although some studies suggest an association between subclinical hypothyroidism and adverse pregnancy outcomes (30–32), this association has not been fully established (33).

Similar to a recent study of a population database including over 184000 women with hypothyroidism (5), we found that hypothyroidism is associated with an increased risk of SPPH. In a study of 702 pregnant women in Pakistan, it was found that PPH was the most common maternal outcome in pregnant women with hypothyroidism (11). However, none of these studies differentiated between overt or subclinical hypothyroidism. Nazarpour et al. found that overt hypothyroidism was associated with an increased incidence of adverse pregnancy outcomes, including PPH, while there is no consensus on subclinical hypothyroidism (33). In this retrospective cohort study of 38439 women from the Third Affiliated Hospital of Guangzhou Medical University, we observed that overt hypothyroidism was an important factor contributing to the elevated risk of SPPH, but subclinical hypothyroidism did not show a significant effect on the development of SPPH. However, Medenica et al. suggest that subclinical hypothyroidism may cause PPH (34). Additionally, treatment of pregnant women with subclinical hypothyroidism using LT4 can significantly reduce the incidence of adverse pregnancy outcomes (35), especially PPH (36). One possible explanation for this discrepancy is the small number of SPPH events among women with subclinical hypothyroidism in our cohort, which may have limited the statistical power to detect a true association. Therefore, for subclinical hypothyroidism, the absence of a significant association in our study should be interpreted with caution rather than as evidence of no risk. Some studies have shown that advanced maternal age is associated with an increased risk of PPH (37, 38). Additionally, Cai et al. found that maternal age ≥ 30 years was related to adverse pregnancy outcomes in pregnant women with hypothyroidism (39). In our study, we observed an age-stratified pattern in the association between hypothyroidism and SPPH. Among participants younger than 35 years, hypothyroidism was identified as a significant risk factor for SPPH. However, for those aged 35 years and older, no significant association was detected. Further examination of the raw data revealed that, within the ≥ 35 years group (n = 8,366), the number of SPPH events among women with overt hypothyroidism (n = 9) and subclinical hypothyroidism (n = 1) was small, which likely limited the statistical power to detect a true association. Thus, the lack of significance in this subgroup should be interpreted with caution.

Thyroid hormone is an important factor in the migration, proliferation, and invasion of trophoblast cells (11). In pregnant women with hypothyroidism, especially overt hypothyroidism, deficiency of thyroid hormones may lead to placental abnormalities (40), particularly placenta previa. Meanwhile, a study (41) showed a higher rate of placenta previa in hypothyroid women (*p* = 0.09). This may the relationship between hypothyroidism and PPH. Another major function regulated by thyroid hormones is mitochondrial energy production and biogenesis (42), and maternal thyroid hormone deficiency may cause PPH by affecting uterine muscle energy production, resulting in weak uterine contractions.

Our study has several strengths, as well as some limitations. Firstly, we selected a large cohort of women from the Guangzhou Medical Centre for Critical Pregnant Women, ensuring a representative sample of critical obstetric patients in the southern region and providing comprehensive data. Secondly, we employed three models incorporating different covariates to explore the relationship between hypothyroidism and SPPH, followed by stratified analysis to identify potential moderating effects of age. Finally, our results remained robust even after accounting for a wide range of potential confounders and performing sensitivity analyses.

The limitations of this study are as follows. First, the observational nature of this retrospective cohort study precludes causal inference. Despite our efforts to enhance robustness through multiple models (adjusted for different covariates), stratified analyses, and sensitivity analyses, the observed association between hypothyroidism and SPPH should not be interpreted as causal and warrants cautious interpretation. Second, hypothyroidism was identified using ICD codes from electronic health records rather than original laboratory values (e.g., TSH and FT4), which may have resulted in diagnostic misclassification. Third, some important clinical information—such as thyroid disease history, prior treatments (e.g., thyroid surgery), and specific medication use—was often scattered across unstructured clinical notes, making systematic extraction unfeasible. As a result, these variables could not be adjusted for in the analysis and may have introduced unmeasured confounding. Fourth, although multiple potential confounders were adjusted for, several key obstetric variables—including induction of labor, chorioamnionitis, and use of oxytocin/uterotonics—were not accounted for, leaving residual confounding a possibility. Finally, this was a single-center retrospective study conducted in China. Variations in iodine intake, thyroid screening practices, and obstetric management worldwide may also limit the generalizability of our findings. Therefore, future prospective, multicenter studies are warranted to validate these results.

## 5 Conclusion

Overt hypothyroidism significantly increased the risk of SPPH, particularly among women aged < 35 years. For subclinical hypothyroidism, the absence of a significant association with SPPH should be interpreted with caution, as the small number of cases of SPPH in women with subclinical hypothyroidism may result in limited statistical power. These findings may offer valuable insights into the relationship between hypothyroidism and SPPH, potentially optimizing maternal outcomes by preventing and intervening in the occurrence of SPPH.

## Data Availability

The raw data supporting the conclusions of this article will be made available by the authors, without undue reservation.
