# Supplementary material for: Differential impact of overt and subclinical hypothyroidism on severe postpartum hemorrhage: a retrospective cohort study
Source: Front Endocrinol (Lausanne). 2025 Nov 5;16:1654856. doi: 10.3389/fendo.2025.1654856 (PMC12626868; doi:10.3389/fendo.2025.1654856)
Supplement: Supplementary file 1 [file Table1.docx]

**Supplementary Table 1.** Reference Ranges of Thyroid Stimulating Hormone (TSH), Free Thyroxine (FT4) in Chinese Pregnant Women at Different Gestational Stages (2012). (1)

| Kit Brands | TSH (mIU/L) | | | FT4 (pmol/L) | | | | Method |
| --- | --- | --- | --- | --- | --- | --- | --- | --- |
|  | Early Pregnancy | Mid Pregnancy | Late Pregnancy | | Early Pregnancy | Mid Pregnancy | Late Pregnancy |  |
| DPC | 0.13–3.93 | 0.26–3.50 | 0.42–3.85 | | 12.00–23.34 | 11.20–21.46 | 9.80–18.20 | Chemiluminescent immunoassay |
| Abbott | 0.03–3.60 | 0.27–3.80 | 0.28–5.07 | | 11.49–18.84 | 9.74–17.15 | 9.63–18.33 | Chemiluminescent immunoassay |
| Roche | 0.05–5.17 | 0.39–5.22 | 0.60–6.84 | | 12.91–22.35 | 9.81–17.26 | 9.12–15.71 | Electrochemiluminescent immunoassay |
| Bayer | 0.03–4.51 | 0.05–4.50 | 0.47–4.54 | | 11.80–21.00 | 10.60–17.60 | 9.20–16.70 | Chemiluminescent immunoassay |

**Supplementary Table 2.** Reference Ranges of Thyroid Stimulating Hormone (TSH), Free Thyroxine (FT4) in Chinese Pregnant Women at Different Gestational Stages (2019). (2)

| Kit Brands | TSH (mIU/L) | | | FT4 (pmol/L) | | | | Method |
| --- | --- | --- | --- | --- | --- | --- | --- | --- |
|  | Early Pregnancy | Mid Pregnancy | Late Pregnancy | | Early Pregnancy | Mid Pregnancy | Late Pregnancy |  |
| DPC | 0.13–3.93 | 0.26–3.50 | 0.42–3.85 | | 12.00–23.34 | 11.20–21.46 | 9.80–18.20 | Chemiluminescent immunoassay |
| Abbott | 0.07–3.38 | 0.34–3.51 | 0.34–4.32 | | 11.30–17.80 | 9.30–15.20 | 7.90–14.10 | Chemiluminescent immunoassay |
| Roche | 0.09–4.52 | 0.45–4.32 | 0.30–4.98 | | 13.15–20.78 | 9.77–18.89 | 9.04–15.22 | Electrochemiluminescent immunoassay |
| Bayer | 0.03–4.51 | 0.05–4.50 | 0.47–4.54 | | 11.80–21.00 | 10.60–17.60 | 9.20–16.70 | Chemiluminescent immunoassay |
| Beckman | 0.05–3.55 | 0.21–3.31 | 0.43–3.71 | | 9.01–15.89 | 6.62–13.51 | 6.42–10.75 | Chemiluminescent immunoassay |
| DiaSorin | 0.02–4.41 | 0.12–4.16 | 0.45–4.60 | | 8.47–19.60 | 5.70–14.70 | 5.20–12.10 | Chemiluminescent immunoassay |
| Tosoh | 0.09–3.99 | 0.54–3.94 | 0.56–3.94 | | 10.42–21.75 | 7.98–18.28 | 7.33–15.19 | Chemiluminescent immunoassay |

**Supplementary Table 3.** Full List of Covariates in Analysis

| Covariates | Explanation |
| --- | --- |
| Age (years) | Age of delivery. |
| Pregestational BMI (kg/m^2^) | Categorized into four groups:  Low weight < 18.5; normal: 18.5–23.9; overweight: 24.0–27.9; and obesity ≥ 28. |
| Education level | The educational level of pregnant women was classified into four groups: college or above, high school or equivalent, middle school, and less than middle school. |
| History of radiation exposure | Maternal history of radiation exposure was recorded as yes, no, and unknown. |
| Previous cesarean delivery | Grouped into no previous cesarean delivery (0), 1–2 previous cesarean delivery, and ≥ 3 previous cesarean delivery. |
| Number of fetuses | Categorized into three groups: singleton, twin, triplet. |
| Gestational age (weeks) | The duration of pregnancy measured in completed weeks from the first day of the last menstrual period or as determined by early ultrasound. |
| Birth weight (g) | The body weight of a newborn infant measured within the first hour of life, before significant postnatal weight loss occurs. |
| Head circumference of newborns (cm) | The measurement of the distance around the largest part of a newborn’s head, usually taken across the forehead and occiput. |
| Stillbirth | Fetal death between 20 weeks of gestation and delivery, or on the time of delivery. |
| Hypertensive disorders of pregnancy | Categorized into gestational hypertension, preeclampsia and eclampsia; gestational hypertension was defined as only if she did not have codes for pre-existing hypertension or preeclampsia or eclampsia. |
| HELLP syndrome | A syndrome characterized by hemolysis, elevated liver enzymes, and low platelet count. |
| Diabetes | Including pregnancies complicated by pregestational diabetes mellitus and gestational diabetes mellitus. |
| ICP | Fasting serum total bile acid (TBA) ≥ 10 μmol/L or random TBA ≥ 19 μmol/L. |
| Anemia | Hemoglobin < 9 g/dL before delivery. |
| Thrombocytopenia | Platelet count < 100^*^10^9^/L. |
| Placenta previa | After 28 weeks of gestation, the placenta is located in the lower uterine segment, with its edge adjacent to or covering the internal cervical os, lying below the presenting fetal part. |
| Placental abruption | The partial or complete detachment of a normally implanted placenta from the uterine wall before the delivery of the fetus, occurring after 20 weeks of gestation. |
| PAS | Including placenta accreta, placenta increta, and placenta percreta. |
| Polyhydramnios | An amniotic fluid volume greater than 2,000 mL observed during gestation. |
| Fetal anomalies | Structural or anatomical characteristics showing significant deviations from normal fetal morphology. |
| Malposition | A cephalic presentation angled more than 45° away from the direct occipito-anterior axis was classified as fetal malposition, as determined by digital examination. |
| Cesarean delivery | Cesarean delivery was defined as childbirth accomplished by surgical incision of the maternal abdominal and uterine walls, rather than by vaginal delivery. |
| Prolonged second stage of labor | Prolonged second stage of labor was defined as more than 1 hour from full cervical dilation to delivery in multiparous women and more than 2 hours in nulliparous women. |
| Fetal distress | A fetal pathophysiological state resulting from inadequate Oxygenation，characterized by atypical fetal heart rate patterns. |
| Abbreviations: BMI, body mass index; ICP, intrahepatic cholestasis of pregnancy; PAS, placenta accreta spectrum. | |

**Supplementary Table 4.** Baseline Characteristics of Study Participants Stratified by Thyroid Status

| Variables | Overall  (n = 34,303) | Hypothyroidism  (n = 33,323) | Subclinical Hypothyroidism  (n = 154) | Overt Hypothyroidism  (n = 826) | *p*-Value |
| --- | --- | --- | --- | --- | --- |
| Age (years) | 31.24 ± 4.78 | 31.21 ± 4.78 | 31.20 ± 4.49 | 32.42 ± 4.66 | < 0.001 |
| Pregestational BMI (kg/m^2^), n (%) |  |  |  |  | 0.468 |
| < 18.5 | 2,164 (6.31%) | 2,105 (6.32%) | 11 (7.14%) | 48 (5.81%) |  |
| 18.5~23.9 | 17,173 (50.06%) | 16,706 (50.13%) | 78 (50.65%) | 389 (47.10%) |  |
| 24.0~27.9 | 10,166 (29.64%) | 9,862 (29.60%) | 47 (30.52%) | 257 (31.11%) |  |
| ≥ 28 | 4,800 (13.99%) | 4,650 (13.95%) | 18 (11.69%) | 132 (15.98%) |  |
| Education level, n (%) |  |  |  |  | 0.332 |
| College or above | 23,484 (68.46%) | 22,844 (68.55%) | 104 (67.53%) | 536 (64.89%) |  |
| High school or equivalent | 4,063 (11.85%) | 3,931 (11.80%) | 17 (11.04%) | 115 (13.92%) |  |
| Middle school | 6,226 (18.15%) | 6,036 (18.11%) | 29 (18.83%) | 161 (19.49%) |  |
| Less than middle school | 530 (1.54%) | 512 (1.54%) | 4 (2.60%) | 14 (1.70%) |  |
| History of radiation exposure, n (%) | 129 (0.38%) | 123 (0.37%) | 0 (0.00%) | 6 (0.73%) | 0.234 |
| Previous cesarean delivery, n (%) |  |  |  |  | 0.037 |
| 0 | 27,561 (80.35%) | 26,745 (80.26%) | 121 (78.57%) | 695 (84.14%) |  |
| 1 | 6,023 (17.56%) | 5,878 (17.64%) | 27 (17.53%) | 118 (14.29%) |  |
| ≥ 2 | 719 (2.09%) | 700 (2.10%) | 6 (3.90%) | 13 (1.57%) |  |
| Number of fetuses, n (%) |  |  |  |  | < 0.001 |
| Singleton | 31,904 (93.01%) | 31,014 (93.07%) | 135 (87.66%) | 755 (91.40%) |  |
| Twin | 2,360 (6.88%) | 2,275 (6.83%) | 18 (11.69%) | 67 (8.11%) |  |
| Triplet | 39 (0.11%) | 34 (0.10%) | 1 (0.65%) | 4 (0.49%) |  |
| Gestational age (weeks) | 37.78 ± 3.30 | 37.79 ± 3.30 | 37.24 ± 3.60 | 37.74 ± 3.16 | 0.115 |
| Birth weight ^a^ (g) | 2,991.74 ± 697.72 | 2,992.36 ± 696.84 | 2,870.42 ± 805.17 | 2,989.28 ± 710.37 | 0.453 |
| Head circumference of newborns (cm) | 32.53 ± 2.79 | 32.53 ± 2.79 | 32.28 ± 2.40 | 32.55 ± 2.57 | 0.522 |
| Stillbirth ^b^, n (%) | 1,096 (3.20%) | 1,073 (3.22%) | 5 (3.25%) | 18 (2.18%) | 0.244 |
| Hypertensive disorders of pregnancy, n (%) | 2,474 (7.21%) | 2,360 (7.08%) | 18 (11.69%) | 96 (11.62%) | < 0.001 |
| HELLP syndrome, n (%) | 54 (0.16%) | 50 (0.15%) | 0 (0.00%) | 4 (0.48%) | 0.083 |
| Diabetes, n (%) | 6,364 (18.55%) | 6,129 (18.39%) | 43 (27.92%) | 192 (23.24%) | < 0.001 |
| ICP, n (%) | 337 (0.98%) | 321 (0.96%) | 1 (0.65%) | 15 (1.82%) | 0.045 |
| Anemia, n (%) | 7,439 (21.69%) | 7,187 (21.57%) | 39 (25.32%) | 213 (25.79%) | 0.008 |
| Thrombocytopenia, n (%) | 213 (0.62%) | 204 (0.61%) | 0 (0.00%) | 9 (1.09%) | 0.198 |
| Placenta previa, n (%) | 1,306 (3.81%) | 1,272 (3.82%) | 3 (1.95%) | 31 (3.75%) | 0.480 |
| Placental abruption, n (%) | 423 (1.23%) | 415 (1.25%) | 2 (1.30%) | 6 (0.73%) | 0.409 |
| PAS, n (%) | 1,078 (3.14%) | 1,045 (3.14%) | 4 (2.60%) | 29 (3.51%) | 0.770 |
| Polyhydramnios, n (%) | 220 (0.64%) | 211 (0.63%) | 0 (0.00%) | 9 (1.09%) | 0.211 |
| Fetal anomalies, n (%) | 502 (1.46%) | 483 (1.45%) | 2 (1.30%) | 17 (2.06%) | 0.350 |
| Malposition, n (%) | 1,793 (5.23%) | 1,728 (5.19%) | 9 (5.84%) | 56 (6.78%) | 0.119 |
| Cesarean delivery, n (%) | 13,682 (39.89%) | 13,217 (39.66%) | 70 (45.45%) | 395 (47.82%) | < 0.001 |
| Prolonged second stage of labor, n (%) | 254 (0.74%) | 243 (0.73%) | 3 (1.95%) | 8 (0.97%) | 0.175 |
| Fetal distress, n (%) | 1,961 (5.72%) | 1,887 (5.66%) | 12 (7.79%) | 62 (7.51%) | 0.042 |
| SPPH, n (%) | 913 (2.66%) | 874 (2.62%) | 5 (3.25%) | 34 (4.11%) | 0.028 |
| ^a^ The weights of twins and triplets were averaged over all fetuses.  ^b^ Stillbirth is defined as the death of at least one fetus.  Abbreviations: BMI, body mass index; ICP, intrahepatic cholestasis of pregnancy; PAS, placenta accreta spectrum; SPPH, severe postpartum hemorrhage. | | | | | |

**References**

1 Endocrinology CSO, Medicine CSOP. A guideline for diagnosis and management of thyroid diseases during pregnancy and postpartum. Chin J Perinat Med 2012;**15**(7): 385-403.

2 Endocrinology CSO, Perinatology CMAA, Association CM. Guideline on diagnosis and management of thyroid diseases during pregnancy and postpartum (2nd edition). Chin J Endocrinol Metab 2019;**35**(8): 636-65.
